# “Making It Work”: A Preliminary Mixed Methods Study of Rural Trauma Care Access and Resources in New Mexico

**DOI:** 10.7759/cureus.11143

**Published:** 2020-10-24

**Authors:** Anna L Carroll, Deanna Garcia, Sandrene J Cassells, Janine S Bruce, Sylvia Bereknyei Merrell, Erika Schillinger

**Affiliations:** 1 Medicine, Stanford University School of Medicine, Stanford, USA; 2 Computer Science, Stanford University, Stanford, USA; 3 Pediatrics, Stanford University School of Medicine, Stanford, USA; 4 Surgery, Stanford University School of Medicine, Stanford, USA

**Keywords:** trauma patients, trauma centers, rural areas, geospatial analysis, qualitative studies, mixed methods research, quantitative and mixed methods research

## Abstract

Introduction

Patients in the rural western United States face challenges accessing trauma and surgical services and are more likely to succumb to their injuries. New Mexico, a rural and medically underresourced state, is a salient space to study these disparities. We examine how travel distance from trauma centers impacts injured patient outcomes and describe care delivery obstacles.

Materials and Methods

We conducted an explanatory mixed methods study by creating geospatial maps of New Mexico’s trauma data, incorporating linear regression analyses on patient outcomes as a function of estimated travel distance from trauma centers. We also conducted qualitative semi-structured interviews with trauma providers to illuminate and provide context for the geospatial findings utilizing a systematic, collaborative, iterative transcript analysis process. We constructed a conceptual framework describing rural trauma care delivery obstacles.

Results

Geospatial analyses revealed that most New Mexicans face long travel times to trauma centers. Comparing regression analyses using different data sources suggests that solely hospital-derived data may undercount rural trauma deaths. Interviews with 10 providers suggest that elements that may contribute to these findings include on-the-ground resource-based challenges and those related to broader healthcare systems-based issues. Our conceptual framework denotes how these elements collectively may impact rural trauma outcomes and proposes potential solutions.

Conclusions

In addressing rural patients’ needs, healthcare policy decision-makers should ensure that their datasets are comprehensive and inclusive. They must also take into account the particular challenges of underserved rural patients and providers who care for them by eliciting their perspectives, as presented in our conceptual framework.

## Introduction

Individuals living in the rural western United States face challenges accessing quality healthcare. Such gaps in access disproportionately affect racial and ethnic minorities, who often live in rural, underserved areas [1]. Trauma, especially severe injuries requiring specialized care and/or surgical intervention, amplifies these disparities [2,3]. These rural patients often live geographically far from care, as described by a 2018 analysis of national hospital emergency general surgery capabilities by Khubchandani et al. [1]. Once involved in a trauma, rural patients facing long travel times to hospitals and often receiving care outside of specialized trauma centers [4] are more likely to succumb to their injuries compared to patients who do not live in rural areas [2-4].

Better characterizing these disparities is the first, crucial step towards improving them. New Mexico is an interesting and relevant space to explore these issues, as it is one of the most rural and medically underresourced states. Two-thirds of its counties are federally designated as both rural and underserved [5]. New Mexico’s rurality impacts trauma care access, as evidenced by Wolf et al.’s 2017 finding that 7 in 10 children involved in fatal motor vehicle collisions there faced a greater than one-hour transport time to any hospital - the highest proportion in the nation - delaying initiation of critical trauma care [6]. As one of the few majority-minority states in the country, more than one million Hispanic/Latino and Native American residents of New Mexico face particular obstacles accessing quality healthcare [7]. Many lack health insurance, with 9.5% of New Mexicans overall self-reporting uninsured status in 2019 versus 8.5% of patients nationally [8]. Based on 2018 data, an even greater portion of these patients of color, 13% of Hispanic/Latino and 21% of Native American nonelderly New Mexicans, are uninsured [9].

In order to improve access to high-quality trauma care throughout the rural United States, rigorous evaluation of the current situation and thoughtful consideration of potential solutions are necessary [10]. Through this preliminary research, we sought to understand whether long travel times and related lack of access to emergency care influence trauma outcomes in New Mexico by creating and analyzing state population maps using publicly available trauma fatality data. We also used qualitative interviews to explore the challenges that rural healthcare providers face delivering care to patients in need. Through our mixed methods approach, where geospatial and qualitative analyses serve to contextualize one another, we present a conceptual framework that richly describes the state of trauma care in rural New Mexico, with the goal of proposing and inspiring solutions that could be applied throughout the rural West to improve trauma care access and outcomes.

## Materials and methods

Overall approach

We used an explanatory mixed methods approach [11] to characterize the state of trauma care in New Mexico, including geospatial (mapping) analysis and qualitative analysis of semi-structured interviews with trauma care providers. We employed this design to construct a conceptual framework on rural healthcare challenges that contextualizes trauma patient outcomes across the state with the experiences and expertise of providers.

Geospatial analysis

We used the 2010 census data [7] to determine the population in each New Mexico county and the American Trauma Society’s “Find Your Local Trauma Center” map [12] to pinpoint the locations of state trauma centers, which are designated as such by the American College of Surgeons (ACS) and/or the State of New Mexico, indicating their level of certification (I, II, or III).

In order to analyze the potential relationship between distance from trauma centers and patient outcomes, we first aggregated fatal unintentional injury data from two publicly available sources: the New Mexico Health Department’s Indicator-Based Information System (NM-IBIS) [13] and the Centers for Disease Control and Prevention’s Web-based Injury Statistics Query and Reporting System (CDC-WISQARS) Injury Prevention & Control Data [14]. We extracted all trauma fatalities from the years 2008 to 2014 (the most recent years for which both data sources recorded trauma fatality outcomes) and noted the average yearly fatalities per 100,000 population for each New Mexico county.

We first explored these data by creating maps through ArcGIS (Environmental Systems Research Institute) [15]. To a map of counties in New Mexico, we added trauma centers coded as level I, II, or III, as points by latitude and longitude. We then displayed different color-coded values on these maps (rural or underserved status, population, and NM-IBIS or CDC-WISQARS fatal injury data).

Our second analytic modality involved using linear regression to assess the relationship between approximate travel time to a trauma center and trauma fatalities. In order to estimate travel time for fatally injured patients living in various counties to trauma centers, we used ArcGIS to create centroid points of all New Mexico counties. We used Google Maps, which takes average traffic and road conditions into account, to calculate the estimated driving time (in minutes) from the centroid point to the location of the closest trauma center (and the state’s only level I trauma center). We plotted the association between estimated driving time (in minutes) from the closest trauma center and trauma fatalities in each county. Note that the county locations cited for unintentional fatal injuries in both data sources are reported based on the decedent’s county of residence (obtained from hospital ICD codes in NM-IBIS and death certificates in CDC-WISQARS), which is presumably but not necessarily where their injury occurred. These databases do not offer any information regarding the location or type of hospital where a patient may have received trauma care, but we assume for the purposes of this analysis that a patient with a life-threatening injury likely would have been taken or transferred to the nearest trauma center or the state’s only level I trauma center. Using R software [16], we fit a linear regression model.

Qualitative study design

We elicited perspectives of New Mexican providers working in various practice environments, along with those of other key experts and informants involved in rural trauma healthcare and research. Consent was obtained in accordance with our Institutional Review Board, which approved this work through their expedited review process.

Qualitative sample

We recruited attending physicians working in emergency medicine and trauma surgery for interviews. Recruitment e-mails were sent to all members of these departments at an academic medical center in New Mexico whose contact information was publicly available on their department websites. We asked recruitment e-mail recipients to share information regarding our study with additional individuals knowledgeable about rural trauma care in New Mexico, who might be interested in participating. This snowball sampling technique allowed for recruitment of providers outside those original institutions (at other sites in New Mexico and the Navajo Reservation). To obtain a more diverse, holistic qualitative interview sample, we also purposefully sought out experts in rural trauma healthcare and research, who had relevant experience in New Mexico or other comparable practice and/or research environments.

Qualitative data collection: semi-structured interviews

We developed an interview guide, partially informed by a theoretical framework identifying patient-perceived barriers in healthcare systems [17]. To elucidate the underpinnings of, and possible solutions to, challenges in rural trauma care, physician participants were asked questions in five topic areas: (1) their professional background, (2) challenges they encounter delivering care to patients in their daily practice, (3) situations involving escalation of care, (4) experiences practicing in urban versus rural settings, and (5) their ideal scope of practice in community hospitals (Appendix I). Follow-up questions varied among interviews based on participant experiences and perspectives. A medical student researcher (A.L.C.) conducted all interviews in person or using secure Zoom virtual conferencing software [18], after each participant signed a consent form, utilizing Zoom for audio recording. A.L.C. transcribed some interviews; the remaining transcripts were obtained through a professional service (Rev.com) and proofread for quality control by A.L.C. prior to analysis.

Qualitative data analysis

We employed a systematic process for interview transcript analysis [19]. A.L.C. inductively developed a preliminary codebook using a single transcript. The analysis team (A.L.C., S.J.C.) discussed the logic and flow of the codebook under the guidance of qualitative research experts (J.B., S.B.M.) to refine it before analyzing a second transcript, with the understanding that additional inductive codes might emerge. With no major changes at that point, we performed an interrater reliability test to ensure that the master codebook was applied consistently (Cohen’s kappa score: 0.76) [19]. A.L.C. coded the remaining eight transcripts using Dedoose software [20], carried out thematic analysis [21], and performed a brief content analysis to categorize which types of trauma services providers prioritize in rural settings.

Conceptual framework construction

Collectively, the themes emerging from the qualitative interview data, in concert with the geospatial analysis results, informed our construction of a conceptual framework describing the challenges faced in providing rural trauma care in New Mexico, along with potential solutions.

## Results

Geospatial analysis results

**Figure 1:**
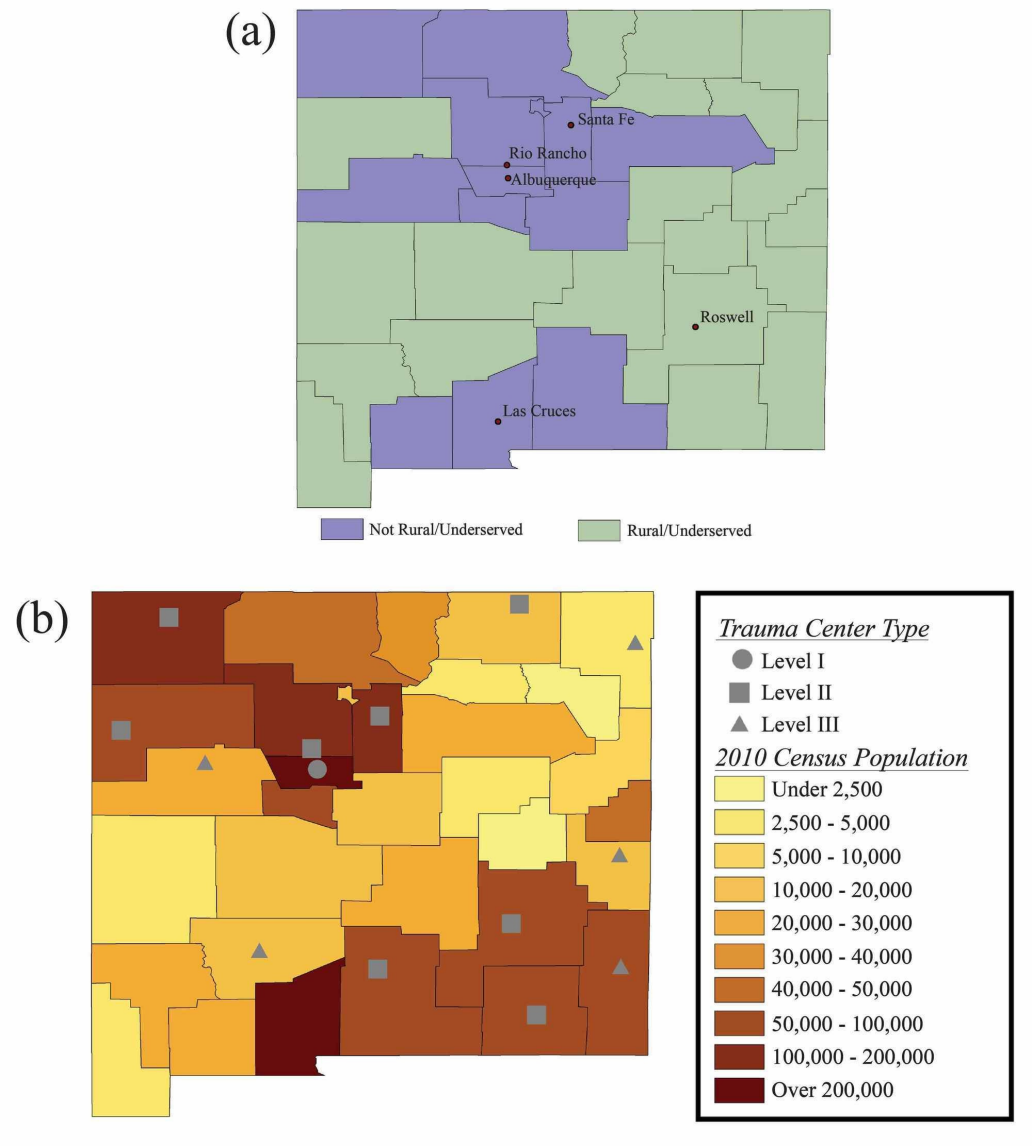
Maps of New Mexico (a) Map indicating the rural and underserved counties in New Mexico, which was created using federal data [5] in ArcGIS [15]. (b) Map including the 2010 census data and indicating the locations of trauma centers of various levels throughout New Mexico, which was created using ArcGIS [15] based on data from the United States Census Bureau [7] and the American Trauma Society [12].

We created maps with federal rural and underserved county designations [5] and 2010 census data [7], indicating the locations of ACS/State of New Mexico-designated trauma centers of various levels [12] throughout New Mexico (Figure 1). Using this second map, ArcGIS [15] analyses determined that approximately one-third (31.4%) of New Mexicans live in counties with an estimated drivetime greater than 1 hour from any trauma center. The majority, or about two-thirds (64.1%), of New Mexicans live in counties, with an estimated drivetime greater than 1 hour from the state’s only level I trauma center.

**Figure 2:**
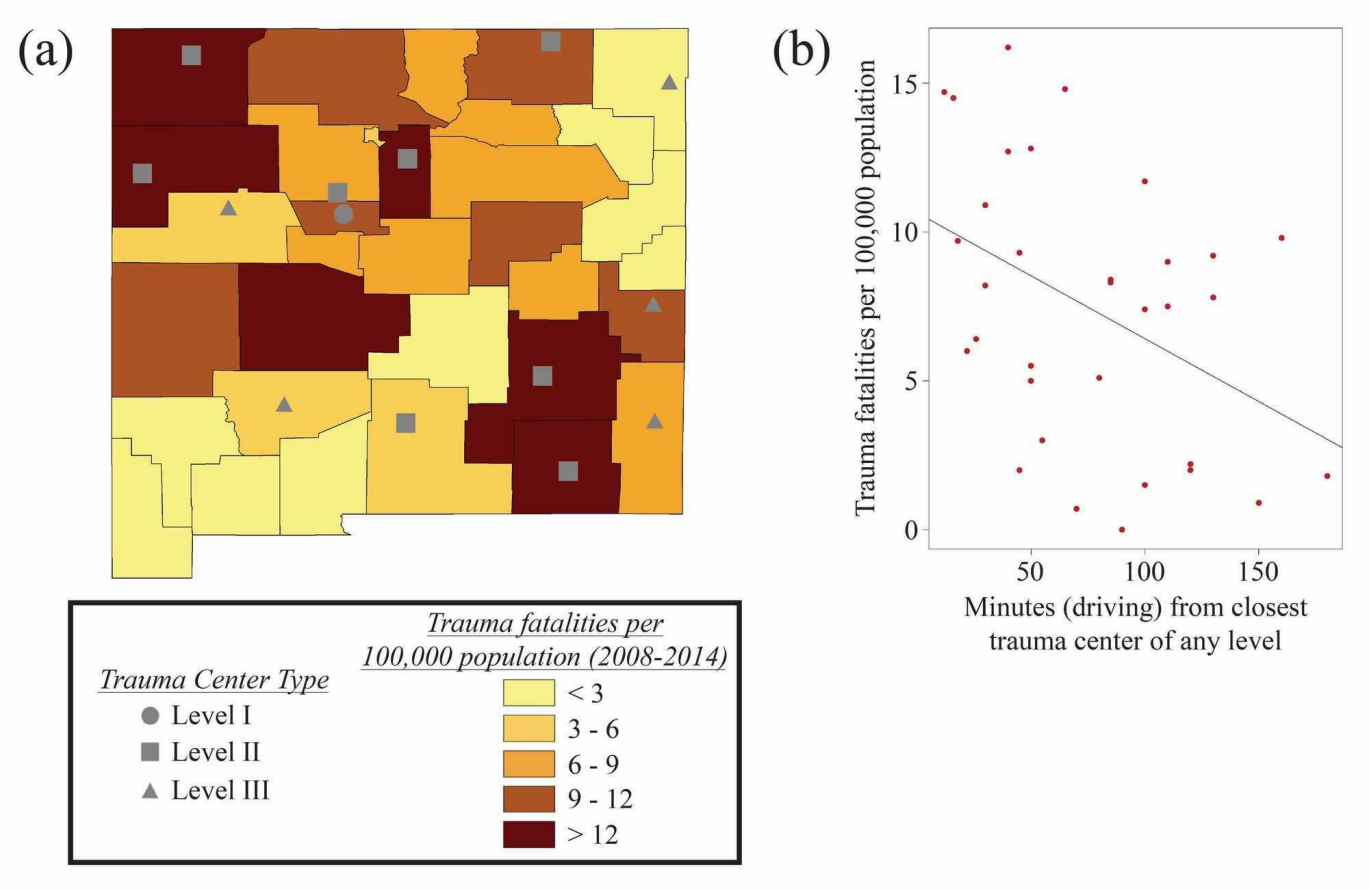
Geospatial Analysis: NM-IBIS (a) Map including NM-IBIS data of trauma fatalities per 100,000 people from 2008-2014 [13] indicating the locations of trauma centers of various levels throughout New Mexico, created using ArcGIS [15] based on data from the United States Census Bureau [7] and the American Trauma Society [12]. (b) A linear regression analysis in R software [16] shows a statistically significant negative association between estimated driving time from the nearest trauma center and trauma fatalities per 100,000 people using only hospital-reported ICD code-derived NM-IBIS trauma fatality data (p = 0.016) [13]. ICD, International Classification of Diseases; NM-IBIS, New Mexico Health Department’s Indicator-Based Information System

**Figure 3:**
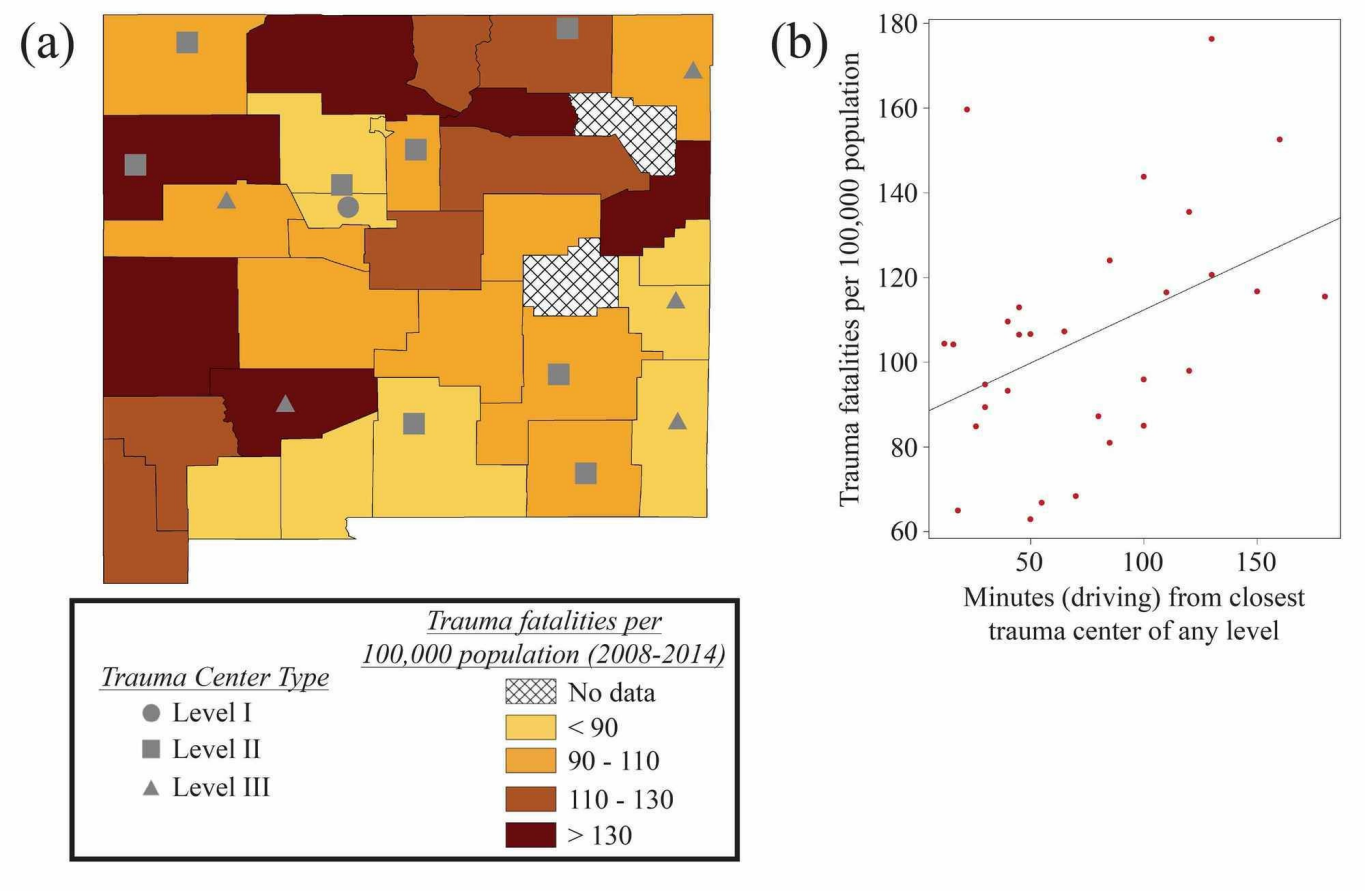
Geospatial Analysis: CDC-WISQARS (a) Map including CDC-WISQARS data of trauma fatalities per 100,000 people from 2008-2014 [14] indicating the locations of trauma centers of various levels throughout New Mexico, created using ArcGIS [15] based on data from the United States Census Bureau [7] and the American Trauma Society [12]. (b) A linear regression analysis in R software [16] shows a statistically significant positive association between estimated driving time from the nearest trauma center and trauma fatalities per 100,000 people using death certificate-based CDC-WISQARS data (p=0.020) [14]. CDC-WISQARS, Centers for Disease Control and Prevention’s Web-based Injury Statistics Query and Reporting System

Utilizing hospital-reported ICD (International Classification of Diseases) code derived data from 2008-2014 (NM-IBIS) [13], we performed a linear regression analysis of trauma fatalities per 100,000 people as a function of estimated drivetime (in minutes) from the nearest trauma center, which unexpectedly revealed a statistically significant inverse relationship (p = 0.016) (Figure 2). Similar examination of CDC-WISQARS trauma fatality data from 2008-2014 [14], which encompasses all unintentional injury-related fatalities reported on death certificates, more predictably showed the opposite relationship. In this case, we found a statistically significant positive association between estimated drivetime to the nearest trauma center and trauma deaths (p=0.020), as well as about ten times more fatalities recorded overall (Figure 3). Note that CDC-WISQARS reports an average yearly national trauma fatality rate of 60 per 100,000 people; New Mexico’s average is higher at 95 per 100,000 people [14].

Qualitative interview participants

We sent recruitment e-mails to 73 Emergency Medicine and Trauma Surgery attending physicians at a large academic medical center in New Mexico; three providers from that institution participated in interviews (response rate = 4.1%), and a fourth nearby providers were referred by one of those participants. A fifth New Mexican participants were recruited through a contact at a second academic medical center in California. There, we purposefully sought five additional participants with expertise and experience providing trauma care or conducting research in New Mexico or similar settings as key informants. The sample included male- and female-identifying physicians with varying academic job titles, levels of seniority, and specialties (Table 1). Individual demographic information (exact geographic location, institutional affiliation(s), departmental position, or personal attributes such as race/ethnicity) is not reported to protect the anonymity of participants.

**Table 1:** Qualitative Interview Participant Demographics

Demographics	New Mexico and Navajo Reservation Providers	Additional Key Informants
Primary practice setting		
Urban	3	5
Rural	2	0
Sustained practice in a rural setting		
Yes	4	1
No	1	4
Specialty		
Emergency medicine (pediatric)	5 (1)	3 (1)
Trauma surgery	0	2
Gender		
Female	2	2
Male	3	3
Years as an attending physician		
≤5 years	2	0
>5 years	3	5
Years at current institution		
≤5 years	2	0
>5 years	3	5

Qualitative interview results: healthcare systems-based challenges

Perspectives from interview participants serving as trauma care providers confirm, contextualize, and explain our geospatial findings. Many participants spoke to the particular challenges of reaching rural trauma patients, potentially leading to poorer outcomes for those individuals injured in remote areas distant from specialized care (Table 2).

**Table 2:** Representative Quotes: Healthcare Systems-Based Challenges Healthcare systems-based challenges in rural settings that trauma care providers report in qualitative interviews, with exemplary quotes reflecting emerging themes.

Representative Quotes (Presented by Theme)
Patient Transfers Are Especially Complex in Rural Settings and Can Limit and Delay Trauma Care
“If someone is really sick and needs a surgical intervention, they potentially could get it here, but ideally they do get it somewhere else...but sometimes they're...too sick to be transferred. Do you provide suboptimal temporizing care or try and risk a transfer, where they can be managed more definitively? That can be tough sometimes.” -Provider working at a rural hospital on the Navajo reservation
“In theory...the way it's supposed to work is that you should...pick up the phone and transfer somebody to an emergency department with a trauma center that...has [a required] resource, but...a lot of these hospitals are overburdened, and they might have spotty coverage from certain...specialists that the patient is going to need.” -Provider working at a rural hospital on the Navajo reservation
“There’s times when non-patient factors do come into the decision making [about patient transfers]...I mentioned the weather...which can increase the time for a crew to arrive. [It’s an issue crossing] state boundaries, especially in the more vulnerable Medicaid population.” - Provider working in urban and rural hospitals in New Mexico
“I have not done this, but...you will talk to doctors who work at smaller institutions, especially rural institutions, who have to call a lot of different hospitals to find an accepting hospital.” - Provider working in an urban hospital in California
“By the time we saw [a patient, it] was about two to two and a half hours after the actual event, or crash...just given the size of New Mexico, it can take quite a bit of time to get patients here...that's one thing that poses unique challenges is the long distances and also the remote locations where some of these traumas occur.” - Provider working in an urban hospital in New Mexico
“UNM is three and a half hours away by driving if you're going fast, and so we try not to send patients there because it's really a burden on those families, and UNM is really overburdened in general. It's the only Level-1 trauma center in the state.” - Provider working at a rural hospital on the Navajo reservation
Trauma Patients Without Insurance Face Barriers To Care
“I see the insurance as the biggest hurdle right now. Because, while you might be initially resuscitated, there's a lot of ongoing care that's required in a bad trauma. It could be...months of rehabilitation and seeing specialists. And if that's not covered...patients may do poorly.” - Provider working in an urban hospital in California
“At [a large academic hospital] we don't take all kinds of insurance because...we're not a state Medi-Cal provider...It's not a matter of [resources]...It’s a matter of, like, the will to provide someone with care based on their socioeconomic status and perceived role in our society.” - Provider working in an urban hospital in California
EMS Plays A Pivotal Role In Trauma Care Access But Varies Widely
“There’s a wide variation that we have to work with in terms of EMS...Within Albuquerque, there’s very highly-trained paramedic crews, and, in some of the rural areas, it may be...all-volunteer.” - Provider working in urban and rural hospitals in New Mexico
“Many of the [Native American] pueblos have their own EMS service, and they will transport their patients to the regional hospitals...It’s another kind of sovereign entity that’s trying to care for their people, but they don’t necessarily have the same hospital resources [as] a city.” - Provider working in urban and rural hospitals in New Mexico
Broader Political Forces Impact Trauma Center Location and Resource Distribution
“Trauma centers sort of happen where they happen. It’s not like somebody says, ‘Well, it makes really good sense for a trauma center to be here. So, we’re going to put one in this area.’ That would be a logical way to do it, but that’s not how it works...hospitals are trauma centers because they raised their hand and said they’d be willing to do it. So, there are places where there really should be one, but there’s not a will to do it.” - Provider working in an urban hospital in California
“States...make decisions around how they...allocate resources, particularly for Medicaid...and...running of the University hospital, which is also the safety net hospital for not just Albuquerque but the entire state...Ultimately, they're political decisions...not based purely on the medical needs.” - Provider working in an urban hospital in California (trained in New Mexico)
“With [national professional organizations]...we...deal with these issues of access...Much of the leadership comes from large urban areas...How do you find out from a rural surgeon...what her needs are?...It’s hard...because they’re usually out in a small community...busy...working, frequently not involved in national politics and advocacy.” - Provider working in an urban hospital in California

Patient Transfers Are Especially Complex in Rural Settings and Can Limit and Delay Trauma Care

Providers expressed frustration at the lack of clear and consistent procedures for transferring trauma patients to a higher level of care. Such challenges were amplified in rural New Mexico, where providers encountered difficulties transporting patients long distances from remote areas to the state’s only level I trauma center, which is often overburdened.

Trauma Patients Without Insurance Face Barriers to Care

Trauma care providers highlighted lack of insurance as a significant challenge impacting patient outcomes. Even when uninsured patients are able to receive initial emergency care after a major injury, they often cannot afford long-term rehabilitation services.

EMS Plays a Pivotal Role in Trauma Care Access But Varies Widely

Physicians working in rural trauma care pointed to the inconsistency in Emergency Medical Services (EMS) availability and capability as a major barrier to reaching trauma patients. Highly trained, professional emergency response teams do not exist in many remote areas, increasing response time to injured patients and limiting their crucial early resuscitation.

Broader Political Forces Impact Trauma Center Location and Resource Distribution

Providers reported that factors outside the sphere of individual healthcare facilities impact accessibility to the appropriate level of care for trauma patients. For instance, determinations regarding where trauma centers are located may not necessarily depend on local need but rather institutional willingness and ability to become certified. Interview participants shared their concerns that hospitals in underresourced areas might not become certified trauma centers because they often do not have the resources to fund all the programs, personnel, and facilities necessary to provide this specified level of care consistently.

Qualitative interview results: healthcare resource-based challenges

In interviews, providers noted that available resources vary widely across different care environments, which may impact patient outcomes in rural, lower-resource settings (Table 3).

**Table 3:** Representative Quotes: Healthcare Resource-Based Challenges Healthcare resource-based challenges in rural settings that trauma care providers report in qualitative interviews, with exemplary quotes reflecting emerging themes.

Representative Quotes (Presented by Theme)
Inpatient Beds Are Often Unavailable, Limiting Hospital Ability to Accept Trauma Patients
“Our hospital runs at 104% capacity at all times.” - Provider working in an urban hospital in New Mexico
“We probably have a similar number of ER visits to [a large East Coast academic medical center], but [it] has two to three times the number of inpatient beds...So, we’re always dealing with an overloaded service and a boarding problem...I have to wonder: are there times where we would’ve observed patients...in the hospital longer...where there...may be a push to get them discharged earlier, just because we don’t have any space?” - Provider working in urban and rural hospitals in New Mexico
“On any given day within a 24-hour span, my hospital will run out of beds...I can't admit people anymore, so they all have to get transferred.” - Provider working at a rural hospital on the Navajo reservation
“The hospital’s full frequently, and so there are times that there are patients that we need to get here that we can’t get here because there’s no place to put them.” - Provider working in an urban hospital in California
Pathways for Rural Trauma Patients Are Unclear When Hospitals Do Not Have Trauma Protocols
“[It’s] easier to deliver good trauma care [when] there is a system in place...One problem I encountered at a [rural] level two trauma center...is that there's not a trauma team...So, it was very difficult to get [trauma] patients admitted to the hospital.” - Provider working at an urban hospital in California
“More than anywhere I've worked...you end up...making up nontraditional plans.” - Provider working at a rural hospital on the Navajo reservation
Lack of Material Resources Limits Ability to Care for Trauma Patients in Rural Facilities
“I’ve worked a shift in [a hospital in Northeastern NM] where the CT scanner was broken, and a part had to be brought from Albuquerque. We had no CT scanner for 24 hours...we ended up doing a lot more transferring than we might have otherwise.” - Provider working in urban and rural hospitals in New Mexico
“Another limitation of the smaller hospitals is the availability of blood...In [a hospital in Northeastern NM], we often only have two units [of] O-negative blood available after which, we’re done.” - Provider working in urban and rural hospitals in New Mexico
Rural Personnel Shortages and High Staff Turnover Rates Impact Trauma Care Delivery
“In [urban California], we're at the trauma center...there's a trauma team and resources...[A] sick trauma patient comes in, and there's sometimes...too many people (laughs) in the room. Whereas, [in rural New Mexico], there was me and [two nurses] and a respiratory therapist....Resources...staffing, turnover, and retention [are] problems out here...a real daily issue...and I'm saying that as someone who's leaving, so (laughs), I'm part of the problem.” - Provider working at a rural hospital on the Navajo reservation
“I received two intubated [pediatric] trauma patients...about 10 minutes apart...But we...just intubated a child...with status epilepticus. We had a resident and a fellow, and [me]. I had to...meet the first trauma patient, and...while I was caring for [them], the fellow [performed] initial evaluation of the second trauma patient. I was going back and forth between the CT scanner and the trauma room and...I think those kinds of situations are challenging just with one ED provider.” - Provider working in an urban hospital in New Mexico
“If [a provider is] the contractor who's not here very often, and they're only here because we don't have enough staff, then, they might not be familiar with all the protocols...You just see delay sometimes, or not as well-organized a response as you'd like, even [if] everything [is] theoretically protocolized and standardized.” - Provider working at a rural hospital on the Navajo reservation
Rural Facilities Often Lack Specialty Services, Limiting the Scope of Trauma Care They Provide
“Rural hospitals...tend to not have certain specialists...I need...neurosurgery, cardiology, GI, ophthalmology...pediatric ICU...ENT...Name a specialty, and, for the most part, I don't have it...essentially every shift I am calling to a capable center to either speak with a specialist for advice, or to transfer a person.” - Provider working at a rural hospital on the Navajo reservation
“New Mexico’s huge...Years ago...someone looked at a job there...I just remember looking their example of pediatric neurosurgery: they had enough work during the daytime for two pediatric neurosurgeons in this large metropolitan area. But, really, who wants to be on call every other night?...It’s just not tenable.” - Provider working at an urban hospital in California

Inpatient Beds Are Often Unavailable, Limiting Hospital Ability to Accept Trauma Patients

Physicians expressed frustration regarding limited hospital space for trauma patients. Many cited an inability for busy centralized, specialty centers to receive incoming trauma patients in need of care escalation. Smaller facilities also cited limited space as one reason they could not manage trauma patients in-house.

Pathways for Rural Trauma Patients Are Unclear When Hospitals Do Not Have Trauma Protocols

Providers reported that many rural hospitals sometimes struggle to manage trauma patients in-house simply because they lack a formal trauma team designated to caring for these patients.

Lack of Material Resources Limits Ability to Care for Trauma Patients in Rural Facilities

Providers working in high-resource academic medical centers acknowledged that they are usually able to coordinate the resources trauma patients require. However, some rural providers encountered limited resource availability.

Rural Personnel Shortages and High Staff Turnover Rates Impact Trauma Care Delivery

Participants noted that it is difficult to recruit and retain rural healthcare providers. In New Mexico, they reported that high rates of turnover can compromise team cohesion and familiarity with trauma protocols, possibly contributing to poorer outcomes for rural trauma patients.

Rural Facilities Often Lack Specialty Services, Limiting the Scope of Trauma Care They Provide

Providers consistently described difficulty recruiting specialists, who almost universally train at urban academic centers, to relocate to and remain in rural areas. Those few specialists who do cover rural areas often endure punishing call schedules, making it difficult to maintain personal lives and support families, contributing to burnout and relocation.

Qualitative interview results: goals and solutions in rural trauma care

Although many barriers exist to “making it work” in rural trauma care environments, as one emergency medicine provider working on the Navajo Reservation described, providers spoke passionately about their ideal vision for rural trauma care and proposed positive steps towards improving outcomes.

Brief Content Analysis: Many Providers Agree on the Ideal Rural Scope of Practice

Most providers agreed on a basic set of emergency services that should be available at any hospital, including airway management and line access. Surgeons added that obstetrics, general surgery, and anesthesia should also be available at most hospitals, with some arguing for certain surgical subspecialties in the community (orthopedics, urology, ENT) and seeing others (neuro, hand, spine, maxillofacial, plastic, and reconstructive surgeries) as more suited to centralized locations. Many providers highlighted surgeons’ procedural case volume to patient outcome relationships as justification. Others spoke to the imprudence of performing invasive interventions in low-resource settings. In an extreme example, an emergency medicine physician in rural New Mexico said, “One [procedure] I struggle with most is...resuscitative thoracotomy because I think it’s certainly within the scope of practice of emergency medicine...but I also think that it’s probably...futile...if there’s not a surgeon who’s there to stabilize that patient after.”

Effective Solutions in Trauma Care Exist, and Providers Propose Additional Ideas

Rural providers reported innovations to overcome practice challenges. One effective solution in neurosurgery, a service not widely available outside the single university hospital in New Mexico, incorporates telemedicine to increase access. Interview participants also suggested the potential utility of a reliable system for coordinating trauma transfers, such as established, protocolized relationships among facilities at different levels of care. However, they noted financial and bureaucratic barriers to establishing and maintaining widespread collaborations.

Importance of Basic Healthcare Access, Resources, and Education for Community Health

Finally, many providers spoke about the importance of access to primary healthcare and basic necessities to maintain overall health and wellness. A California provider mentioned the “social determinants of health,” saying, “That stuff all plays into health much more than the medical care.” Interview participants also pointed to the importance of promoting community education (such as use of seatbelts) and preventive measures (such as distributing and promoting the use of bicycle helmets and car seats), in addition to providing trauma care. One New Mexico provider explained that “if we can prevent injuries from occurring in the first place, I think that would ease the burden on the system and promote a healthier community.”

Conceptual framework on rural trauma outcomes

**Figure 4:**
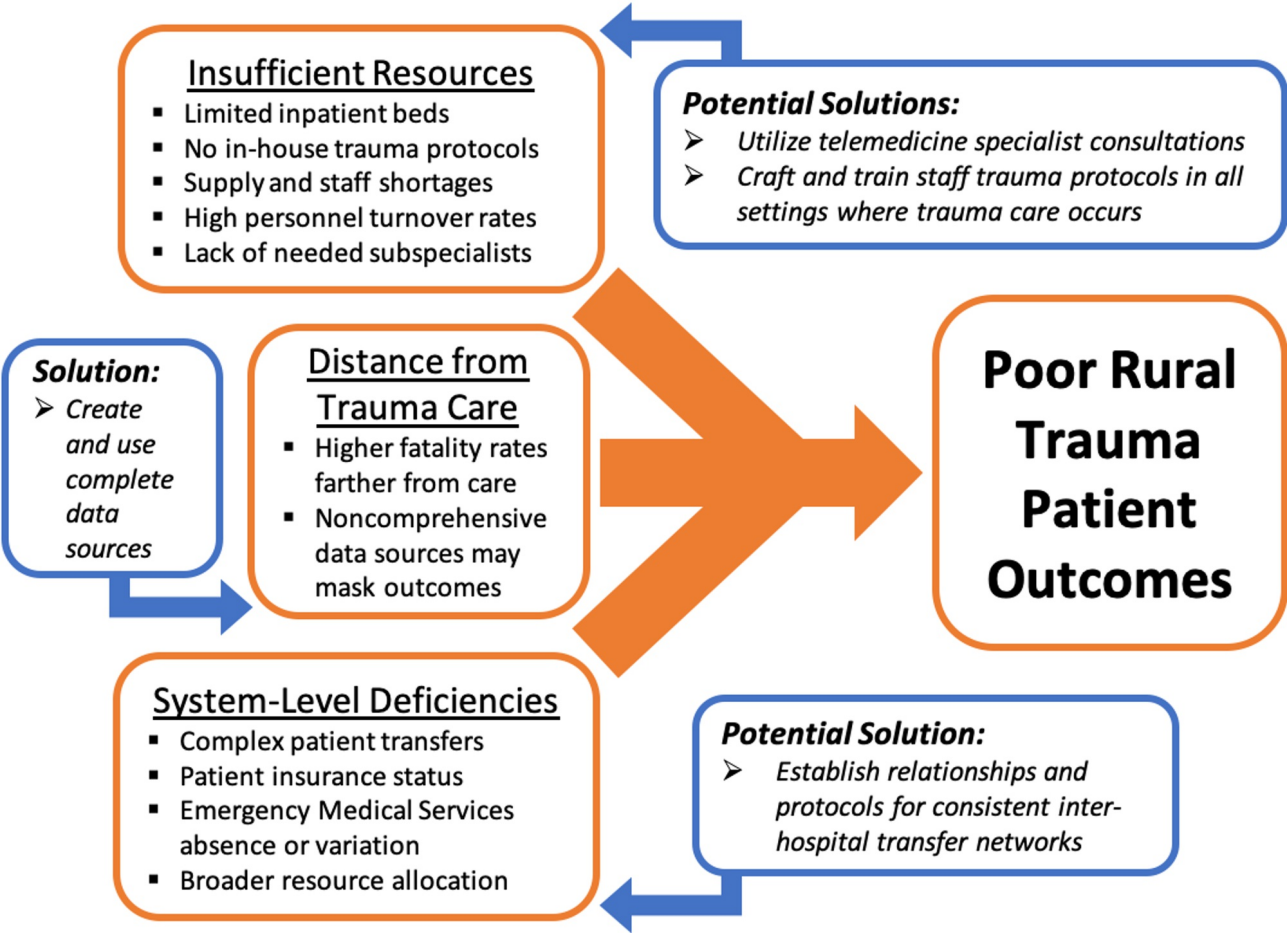
Conceptual Framework on Rural Trauma Outcomes Conceptual framework depicting the factors that may lead to poor outcomes for rural trauma patients, derived from geospatial analysis of New Mexico trauma fatality data and qualitative interviews with trauma care providers and experts. Collectively, long travel distances to sites of definitive care, along with personnel and material resource shortages and deficiencies in healthcare protocols and systems, might limit patient access to trauma care and facilitate suboptimal care delivery in rural settings. Healthcare decision-makers should incorporate comprehensive outcome datasets and take into consideration the perspectives of on-the-ground providers and experts so as to not overlook particular needs in rural environments. This conceptual framework proposes some potential pathways to overcome these challenges and could serve as a tool to help inform and inspire further solutions.

This conceptual framework (Figure 4) derived from our geospatial and qualitative interview data describes the interactions among the challenges of travel distance, resource shortages, and healthcare system inefficiencies in delivering accessible, high-quality trauma care to rural patients. The framework points to potential solutions and innovations that our interview participants shared, which may help inform public health policy efforts to enact reforms.

## Discussion

The results of, and conceptual framework derived from, this preliminary mixed methods study using geospatial mapping and qualitative analysis paint a multi-faceted picture of trauma care access and resources in rural New Mexico. In this case, distance from care emerged as one important access barrier warranting comprehensive investigation.

Our first mapping analysis using hospital ICD code-derived trauma fatality data compiled by the New Mexico Health Department (NM-IBIS) [13] showed that patients living in counties closest to the state’s trauma centers had the poorest trauma fatality outcomes. While the needs of vulnerable patients in these largely urban areas cannot be dismissed, we found this first set of results surprising because it seemed to contradict previously demonstrated national trends indicating that increased distance from trauma centers negatively impacts patient outcomes [2]. This trend of poorer trauma patient outcomes at a farther distance from sites of care has been similarly observed and further validated in international settings as well [22]. In contrast to our first analysis and in line with the literature, our second analysis using CDC-WISQARS data [14], which includes all trauma fatalities (gathered from death certificates) occurring both within and outside of hospital and EMS systems, demonstrated that expected trend wherein patients living farthest from care in largely rural areas had the poorest outcomes.

Clearly, our geospatial analysis results demonstrate that all trauma data sets are not created equal. While hospital-derived databases are crucial for healthcare research, our findings highlight the important understanding that they may undercount rural patients. In other words, they are limited to reporting patients who were capable of reaching care in the first place or remained stable enough for transfer from the field. When we included patients in our analyses who succumbed to their injuries before reaching care, although some may have died immediately at the time of their injury with no opportunity for intervention, for many others, distance appears to be a major obstacle, predicting increased trauma mortality for patients injured further from trauma centers.

Our qualitative findings corroborate this interpretation of our results. One interview participant, a Pediatric Emergency Medicine physician and researcher at a large academic medical center in California, succinctly described the challenges faced during this study:

“When you're trying to look at outcomes, the issue is the distance traveled. So, if somebody is really, really, really sick, they might die before they have a chance to transfer...It's really hard when you look at these administrative data sets...to see outcomes, especially because [they are] limited to...procedures...and things like that. So, they're not true clinical outcomes.”

Our mixed methods approach using two datasets and interviews allowed us to gain more nuanced and essential perspectives on the realistic impact of travel distance on trauma outcomes.

An important implication of these results is that decision-makers in government agencies and hospital administration must understand the source of the data on which they are conducting analyses informing crucial decision-making, such as funding and other resource allocation. If these players utilize trauma data exclusively collected in hospitals based on diagnostic codes that do not capture trauma fatalities occurring outside healthcare facilities, the picture on which they are basing their policy decisions may be incomplete, possibly failing to fully recognize rural healthcare problems and needs.

Qualitative analysis of interviews with trauma providers reaffirmed our findings that physical distance from care is a crucial factor and revealed additional forces contributing to rural patient healthcare access and clinical outcomes. Physicians described in-house logistical challenges plus unique stresses of rural environments, such as complex patient transfers and personnel and resource shortages, along with a lack of protocolized care. Some emergency medical physicians interestingly spoke of the paramount importance of primary care, preventative health interventions, and community education in rural health, sentiments corroborated by previous analyses, such as in the case of booster seat use [23]. They consistently pointed to health insurance, which many New Mexicans struggle to access [8,9], especially those in rural areas living far from care, as a related key factor determining trauma patient outcomes. This assertion is consistent with existing literature demonstrating that trauma outcomes are poorer for patients without health insurance at the time of their injury [24,25].

Our findings point to solutions to some of these challenges, particularly in the realm of telemedicine, which, as past studies indicate, patients are open to utilizing [26], and several examples of implementation demonstrate streamlined care and improved outcomes [27]. Furthermore, standardizing protocols through designation of more rural hospitals as trauma centers [4], or by training staff in Advanced Trauma Life Support (ATLS) protocols, could help mitigate issues of high turnover. These programs must be implemented in ways that are not cost-prohibitive or cumbersome to the operations of already underresourced facilities. Overcoming challenges to increasing rural healthcare access by implementing these kinds of innovations, informed by comprehensive data and local provider and expert perspectives, will be crucial to improving trauma outcomes for patients everywhere.

Limitations

Geospatial Data Limitations

In our geospatial analyses, we used estimated patient driving time from trauma centers as our proxy for access to care. However, the use of airlifting in some areas where rural EMS work, albeit a service that takes considerable time and resources to coordinate, may have been a confounding factor.

Furthermore, using geographic centroids of counties as the points of origin in our driving time calculations both assumes that patients sustained injuries within their county of residence and does not represent an actual driving time from any given patient’s injury location to relevant trauma centers or other locations where they received care. However, all methods of computing such distances introduce inherent error [28], and literature precedent exists for utilizing centroids to measure the approximate distance from an area to the nearest available healthcare resources [29]. Future analysis could be refined by using population-weighted centroid points to more accurately reflect distance from care on a per-county basis or by creating datasets that report comprehensive trauma outcome data on a more granular geographic scale.

We also chose to include only trauma centers located within the state of New Mexico in our analysis, possibly failing to take into account other hospitals where trauma care for New Mexican patients occurs. Our choice was based on the assumption that patients with life-threatening injuries should receive care at a specialized trauma center. This idea was corroborated by qualitative interview participants with experience in both transferring and receiving facilities, as well as previous analyses concluding that outcomes are superior for trauma patients treated in certified trauma center settings [4]. More comprehensive further analysis should include hospitals in New Mexico providing surgical trauma care that may not be trauma centers, as well as nearby hospitals across state lines that serve New Mexicans.

Additionally, in this preliminary study, we used accidental trauma fatality as a logical endpoint for our study, but more comprehensive analyses including non-accidental trauma fatality and overall morbidity information would have been valuable. A limited number of years of trauma outcome data were available, and we were working with small numbers of cases and outcomes overall due to the small population of the rural state of New Mexico, limiting generalizability.

Potential Qualitative Sample Biases

Sample size limitations also apply to the qualitative interview participants, who volunteered for the study in a manner introducing self-selection bias. A power dynamic also existed between the medical student researcher conducting the interviews and the attending physician participants, some of whom are faculty members at the student’s home institution. Nonetheless, thematic saturation was reached, pointing to the richness available in even small qualitative samples [30] and suggesting opportunities for further study.

While the inclusion of key informants practicing outside New Mexico created a rich and well-rounded qualitative sample, it would have been ideal to include more providers from a wider diversity of trauma care settings across New Mexico. Particularly, due to the limited number and busy services of trauma surgeons currently practicing in New Mexico, none could be recruited for this research, leading us to intentionally recruit surgeons with relevant knowledge and past experience practicing in New Mexico or in other comparable settings. Understanding rural trauma surgeon perspectives, as well as engaging local interest groups like the New Mexico Emergency Medical Services Trauma Care Program in the state’s Department of Health, is crucial to fully characterize the state of trauma care. Collectively, these attributes temper our results and limit generalizability. Nonetheless, our small sample does include reasonably comprehensive perspectives from emergency medicine physicians working at New Mexico’s only level I trauma center, as well as three separate sites across rural parts of the state and the Navajo Reservation.

## Conclusions

This preliminary study offers insight into the state of trauma care in the rural and underserved setting of New Mexico using a mixed methods design. Through geospatial analysis, we outlined the limitations of hospital-reported trauma fatality data, wherein a comprehensive picture of trauma patient outcomes shows increased mortality at a farther distance from care. Qualitative analysis of interviews with trauma care providers and experts confirmed these findings and outlined numerous reasons why trauma outcomes may be poorer for rural patients. These interviews revealed in-house resource deficits and healthcare systems based obstacles to delivering comprehensive trauma care, with many stresses particular to rural environments. Overall, our results informed creation of a conceptual framework describing obstacles to rural trauma care delivery applicable to healthcare decision-makers. Namely, our findings point to the need for policy makers to understand the source of their data, ensuring that it includes comprehensive reporting of outcomes such as trauma fatalities, so as not to inadvertently overlook the needs of rural patients. They should also include the vital perspectives of providers working on the ground and other experts when designing and implementing healthcare policy to best serve all patients. Our study brings together geospatial data and systematic analysis of trauma care provider perspectives to present a conceptual framework that more completely describes healthcare challenges in service of rural patients’ needs. Our hope is that this framework can serve as a tool for decision-makers as they endeavor to overcome complex challenges in rural healthcare.

## Appendices

Appendix I. Qualitative Interview Guide

**Table 4:** Qualitative Interview Guide Questions utilized to prompt discussion during qualitative interviews with trauma care providers and experts.

INTRODUCTION: Thank you so much for taking the time to sit down and chat with me. As a medical student at [INSTITUTION] interested in pursuing a career in surgery, who grew up in rural New Mexico, I am passionate about issues of healthcare access in the rural West. I really appreciate your perspectives as we work to overcome complex challenges in this area. I am going to ask you a few questions about your background and experiences, as well as your opinions about healthcare and patient access to surgical services more broadly. Your answers will help us understand how to most efficiently allocate resources and better serve patients across the rural West. As stated on your consent form, your responses will be de-identified, but, where applicable, they may be tied to the geographic region where you practice when we present results of this study. This session is being recorded on two secure, encrypted devices (this laptop and this iPad). Copies will be retained on those devices and the [INSTITUTION] Box pending transcription, after which they will be deleted, and transcripts will be retained in those same secure locations. Do you have any questions for me before we get started? TRANSITION: Let’s start by learning a bit about your background.	
1) Background: Please discuss your journey to trauma care and/or surgery and your decision to practice at [YOUR PRIMARY INSTITUTION].	Possible Prompts: Where are you from? Where did you train? What drew you to trauma care and/or surgery? What is your favorite part of your work?
TRANSITION: Now, I’d like to talk a bit more about your day to day work.	
2) Challenges: I’d like to discuss the challenges you encounter delivering care to all patients in need on a typical day at work, if any. Can you please tell me about a time when administering care that a patient needed was difficult? Did you overcome this challenge? If so, how? GO TO EXAMPLES (LEFT) UP FRONT, IF NEEDED	Possible Prompts: Possible areas of discussion: “challenges or barriers in the area of access to care for patients; challenges or barriers in the area of in-hospital care for patients; and challenges or barriers in the area of governance or health policy.” Do you think that any patient who needs your care has access to it?
3) Escalation of Care: Now, I’d like to know what you do if you ever encounter a patient requiring a higher level/different type of care than you can directly provide, for example, a surgical subspecialty. How is that patient referred and/or counseled?	Possible Prompts: How do we prevent such a situation happening again in the future? How do we better connect patients without local options for care to other resources? Should large academic medical centers, like [INSTITUTION], play a role in providing surgical care to patients from rural areas of the United States? If so, what would that look like?
TRANSITION: At this point, I would like to hear about your experiences outside of [YOUR PRIMARY INSTITUTION].	
4) Rural versus Urban Practice: Since you primarily work at [INSTITUTION], I imagine you typically practice in an [URBAN/RURAL] environment. Oppositely, have you ever worked in an [URBAN/RURAL] setting? à If so, where? How did those experiences at [YOUR PRIMARY INSTITUTION] and [THIS OTHER SETTING] differ? à If not, what have you not worked in such a context?	Possible Prompts: Again, mention three areas of challenges (above), and how they might compare and contrast in each practice context. Would you ever consider practicing in the opposite context? Why or why not? What rural versus urban differences would you expect in the kinds of challenges providers face?
TRANSITION: Finally, the last topic I’d like to discuss may or may not pertain to your practice, in particular. Rather, I’d like to hear what you have to say about a few broader ideas in healthcare access.	
5) Scope of Practice: What surgical services are necessary for a community to be safe and healthy?	Possible Prompts: What’s an example of a service that should be accessible at any hospital? What types of procedures should rural surgeons be trained to perform? Please give an example of a procedure that should only be performed at a highly-specialized, tertiary/quaternary care center (like [INSTITUTION])?
CONCLUSION: Is there anything else you’d like to share on these topics that we did not cover? Would you like to receive a summary of the results of this study? Thank you for your time and remember you can contact me with any questions or concerns.	
